# Cap Polyposis—A Rare Cause of Rectal Bleeding in a Young Woman

**DOI:** 10.14309/crj.0000000000001423

**Published:** 2024-07-10

**Authors:** Ângela Domingues, Ricardo Araújo, Nuno Dias, Américo Silva

**Affiliations:** 1 Department of Gastroenterology, Centro Hospitalar Tondela-Viseu, Viseu, Portugal; 2 Department of Pathology, Centro Hospitalar Tondela-Viseu, Viseu, Portugal

**Keywords:** cap polyposis, inflammatory pseudopolyps, cloacogenic polyps, mucosal prolapse syndrome

## Abstract

Cap polyposis is a very rare clinical entity, with only few cases reported in the literature. It is a benign disease, characterized by inflammatory polyps of the gastrointestinal tract. A 35-year-old woman was referred to our Gastroenterology department due to rectal bleeding. Endoscopic examination revealed multiple polypoid lesions in the middle/lower rectum. Histology was compatible with cap polyposis. She underwent mucosectomy of the lesions, with clinical and endoscopic improvement. Clinical findings and therapeutic approach represent a challenge in this pathology.

## INTRODUCTION

Cap polyposis is a clinical entity first described more than 20 years ago, although it is still a very unrecognized disease. Few cases have been described worldwide, and many are misinterpreted as an inflammatory bowel disease, leading to prolonged and ineffective treatments. Given its rarity, the clinical findings and therapeutic approach remain a challenge.

## CASE REPORT

**Figure 1.**
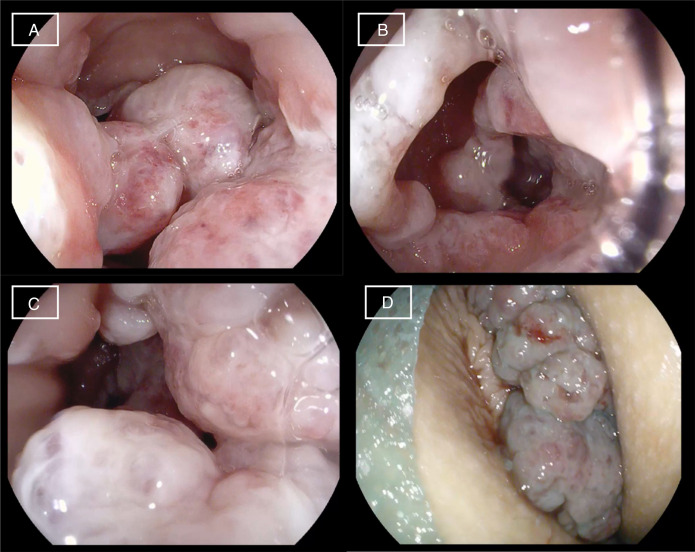
Ileocolonoscopy. Multiple polypoid lesions in the middle/lower rectum, covered by fibrous mucus (A, B, and C). Multiple large polyps, located at the anorectal junction, and prolapsing exteriorly through the anal canal (D).

**Figure 2.**
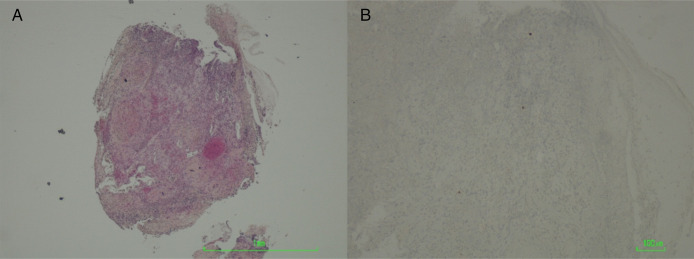
Rectal biopsies. Granulation tissue, without identifiable epithelium or neoplasia (hematoxylin-eosin) (A). Immunostaining for cytomegalovirus (B). (A) 40×; (B) 200×.

**Figure 3.**
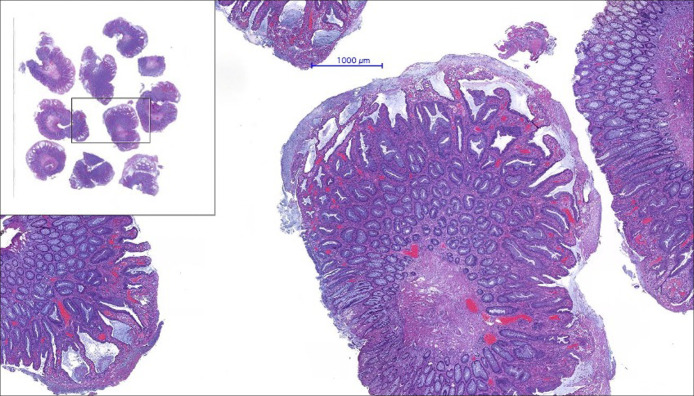
Polypectomy (hematoxylin-eosin, 40×). Multiple colonic polyps (inset) with glandular hyperplasia, dilation, and elongation, accompanied by extensive superficial erosion, fibrinogranulocytic exudate, and mucus cap (cap polyposis).

**Figure 4.**
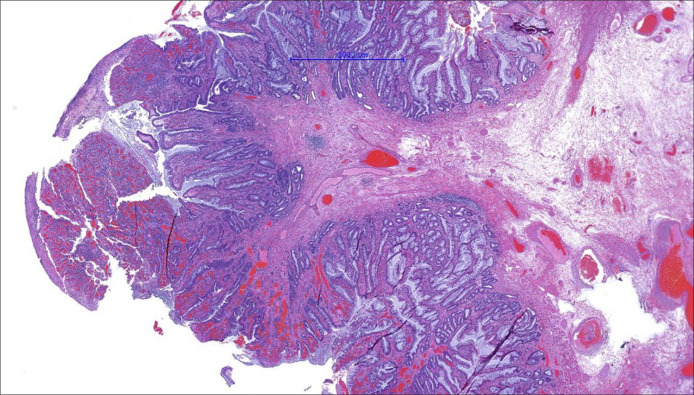
Rectal polyp (hematoxylin-eosin, 60×). Polypoid lesion with verticalization of muscularis mucosa muscle fibers and accompanying dilated venous blood vessels, suggesting a prolapse-type lesion of the juxta-anal rectal mucosa. In this section, partial erosion is identified.

**Figure 5.**
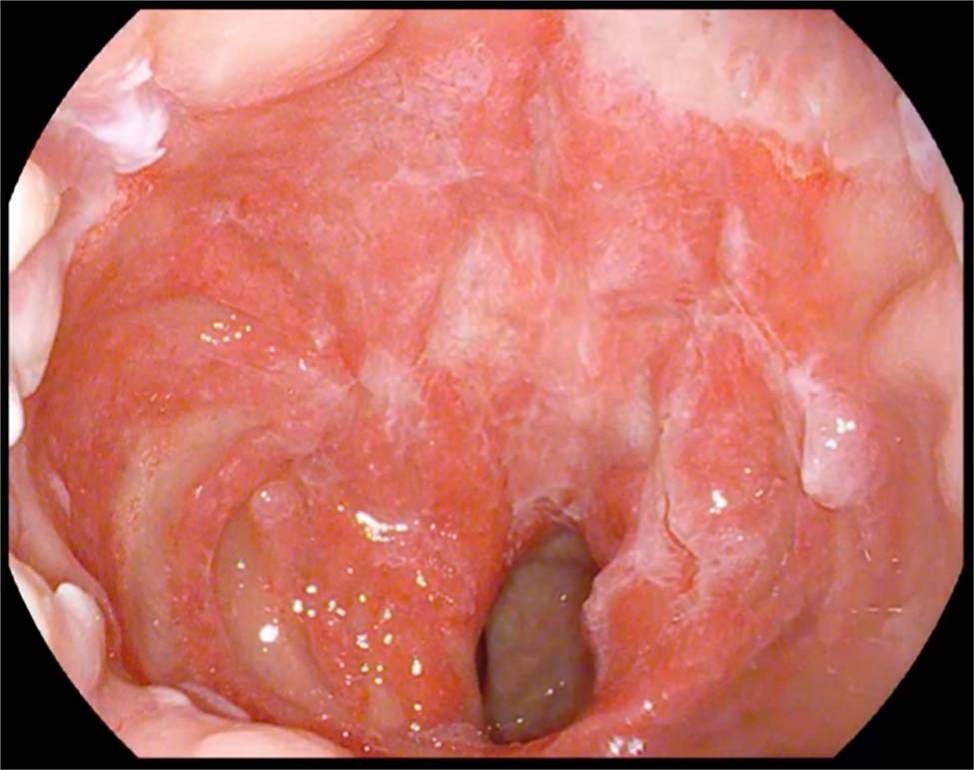
Retossigmoidoscopy. The mucosa presents a scarring pattern in relation to previous endoscopic therapy, but without evidence of recurrence.

A 35-year-old woman was referred to our Gastroenterology department due to rectal bleeding, anemia, and a rectal mass on digital examination. She had an unremarkable medical history besides chronic constipation. She was submitted to an ileocolonoscopy, with the identification of multiple polypoid lesions in the middle/lower rectum and anal canal (Figure 1). Biopsies revealed inflammatory pseudopolyps, absence of malignancy, and focal positive immunohistochemical stain for cytomegalovirus (CMV) (Figure 2). Serology for CMV demonstrated a positive test for IgG and negative for IgM. She was treated with intravenous ganciclovir without significant improvement of symptoms. Blood analysis revealed severe anemia and hypoalbuminemia. Electrolytes, liver enzymes, immunoglobulins, complement, autoantibodies, calprotectin, inflammatory markers, and tumoral markers were within the reference range. *Chlamydia trachomatis, Neisseria gonorrhoeae*, HIV, *Treponema pallidum*, and hepatitis B and C were negative. Table 1 presents a summary of the laboratory and infectious panel. An abdominal and pelvic computed axial tomography scan was performed, describing a thickened rectal wall without other abnormalities. Upper endoscopy was normal, and gastric biopsies were positive for *Helicobacter pylori.* Eradication therapy was prescribed, and a following urea breath test was negative. Four months after, colonoscopy was repeated to perform biopsies which revealed an ulcerated proctitis without malignancy and without positivity to CMV or other infections pathogens. The patient presented with clinical deterioration, with frequent rectal bleeding, severe anemia treated with red cell blood transfusion, and persistent hypoalbuminemia. At that point, it was decided to proceed to the endoscopic removal of the majority of the rectal polyps with mucosectomy and the lesions of the anal canal with transanal excision surgery. Histology identified elongated serpiginous crypts and a “cap” of inflammatory granulation tissue with fibrinopurulent exudate, compatible with “cap polyposis” (Figure 3). The anal canal lesions were classified histologically as inflammatory cloacogenic polyps (Figure 4). At 2-month follow-up, she was almost asymptomatic, her hemoglobin levels normalized and with no endoscopic relapse (Figure 5).

**Table 1.** Laboratory findings and infectious panel

	Test	Result
Blood and fecal analysis	HIV 1 + 2	Negative
	*Treponema pallidum*	Negative
	CMV IgM	Negative
	CMV IgG	Positive
	Epstein-Barr IgM	Negative
	Epstein-Barr IgG	Positive
	*Toxoplasma gondii* IgM	Negative
	*Toxoplasma gondii* IgG	Negative
	Hepatitis A IgM	Negative
	Hepatitis A IgG	Positive
	Hepatitis B—antigen HBs	Negative
	Hepatitis B—antibodies HBs	Negative
	Hepatitis B—antibodies HBc	Negative
	Hepatitis C—total antibodies	Negative
	Calprotectin	55 μg/g (normal range <150)
	ASCA	Negative
	ANCA	Negative
	CEA	0.5 ng/mL (normal range <3)
	ESR	6 mm (normal range 0–20)
	Immunoglobulin A	103 mg/dL (normal range 40–350)
	IgG	760 mg/dL (normal range 650–1,600)
	IgM	54 mg/dL (normal range 50–300)
Biopsy from rectal samples	*Chlamydia trachomatis* real-time CRP	Negative
	*Treponema pallidum* real-time CRP	Negative
	*Neisseria gonorrhoeae* real-time CRP	Negative
	CMV DNA real-time CRP	Positive
	Herpes simplex 1 DNA real-time CRP	Negative
	Herpes simplex 2 DNA real-time CRP	Negative

ANCA, antineutrophil cytoplasmic antibodies; ASCA, anti-saccharomyces cerevisiae antibodies; CEA, carcinoembryonic antigen; CMV, cytomegalovirus; CRP, C-reactive protein; ESR, erythrocyte sedimentation rate; HBc, hepatitis B core; HBs, hepatitis B surface; IgG, immunoglobulin G; IgM, immunoglobulin M.

## DISCUSSION

Cap polyposis is a benign disease of the colon characterized by inflammatory polyps, typically located at the apex of mucosal folds. It was described for the first time in 1985,^1^ and since then, less than 100 cases have been described worldwide.

This disease is more frequent in adult patients, but there were case reports in pediatric patients. The median age at diagnosis is 52 years, and it has a slight female preponderance.^1,2^

The most important differential diagnoses are malignant neoplasms of the gastrointestinal tract and inflammatory bowel disease. Ulcerative colitis is the most common misdiagnosis, which can lead to prolonged and ineffective therapy.^1–3^

The etiological mechanism of the disease is unknown, although mechanical, inflammatory, or infectious causes have been proposed.

This clinical entity presents histopathological similarities with prolapse mucosal syndrome, and it is proposed that it may derive from an intestinal motility disorder. Prolapse of the rectal mucosal folds, secondary to excessive straining at defecation, can lead to the appearance of polypoid lesions and their mucous lining. The fact that these polyps are more common in the distal colon, the symptomatic improvement after avoiding severe straining at defecation and the high prevalence of constipation in this group of patients support this mechanical etiological hypothesis.^4,5^

An infectious cause is proposed as a possible etiological factor. Cases of regression have been described after antibiotic therapy with metronidazole in monotherapy. There is a strong association between “cap polyposis” and *H. pylori* infection, although the bacteria has never been detected in the colonic mucosa. This finding raises the question of whether eradication therapy influences reduction in lesion burden by direct elimination of the bacteria or whether this occurs as an indirect effect on intestinal microbiota.^6,7^ One case report demonstrated that antibiotic treatment led to regression of cap polyposis through significant alterations in fecal microbiota composition and that microbiome-targeted therapy may be useful in this disorder.^8^

There are no specific diagnosis criteria. It is based on clinical, endoscopic, and histological findings. The most common symptoms are mucous discharge, diarrhea, rectal bleeding, abdominal pain, and tenesmus.^1^ Serum albumin levels are often low, and it has been described an association with protein-losing enteropathy. There are 2 cases in the literature associating cap polyposis with this entity, with one of them suggesting a protein loss directly from the polyps. The diagnosis was made with technetium-labeled human albumin scintigraphy, and it has been considered the gold-standard investigation.^5,9,10^ Serum inflammatory markers are usually within reference values, and extraintestinal manifestations are rare.

The typical endoscopic appearance is the presence of polypoid lesions coated with a fibrinopurulent exudate. Polyps can vary in size, morphology, location, and number.^1^ The most common location is within the distal portion of the gastrointestinal tract, mostly the sigmoid colon and rectum.^9^ However, gastric and proximal colon locations have also been described, including proximal extension of a distal localized disease.^11–13^ Polyps present histologically with hyperplastic, tortuous, elongated crypts with an inflammatory infiltrate in the lamina propria. The surface is ulcerated and covered with a fibrinopurulent exudate.^1^

There is still no well-established therapeutic approach since the etiology remains undetermined. The use of corticosteroids, aminosalicylates, infliximab, and antibiotics, such as metronidazole, has been described. *H. pylori* eradication therapy is also recommended when positive.^2,11,14,15^ Endoscopic resection of polypoid lesions seems to be the most effective therapeutic measure in controlling symptoms, although recurrence is frequent. The most used technique is mucosectomy, although there is a reported case of submucosal dissection.^7^ In refractory cases, surgical resection of the affected colon or even total colectomy may be considered.^1^ Patients should implement behavioral modification strategies, such as the use of laxatives and avoidance of straining at defecation.^14^

Cloacogenic polyps are benign lesions that belong to the “mucosal prolapse syndrome” and are therefore associated with excessive straining at defecation. It comprehends multiple clinical entities such as solitary rectal ulcer syndrome, prolapsing mucosal polyps, and cap polyposis.^16^ They are typically found at the anorectal junction, although they can be identified throughout the colon.^17^ Unlike cap polyposis, cases of malignant transformation have been described.^18^

## DISCLOSURES

Author contributions: Â. Domingues, R. Araújo: literature research; Â. Domingues: manuscript preparation; Â. Domingues, R. Araújo, N. Dias: manuscript supervising and editing; All authors revised, reviewed, and approved the final version; Â. Domingues is the article guarantor.

Financial disclosure: None to report.

Informed consent was obtained for this case report.
